# Review and Analysis of Technical Designs of Rear Underrun Protective Devices (RUPDs) in Terms of Regulatory Compliance

**DOI:** 10.3390/s22072645

**Published:** 2022-03-30

**Authors:** Mirosław Gidlewski, Jerzy Jackowski, Paweł Posuniak

**Affiliations:** 1 Military University of Technology (WAT), ul. gen. Sylwestra Kaliskiego 2, 00-908 Warsaw, Poland; miroslaw.gidlewski@wat.edu.pl (M.G.); jerzy.jackowski@wat.edu.pl (J.J.); 2 Łukasiewicz Research Network-Automotive Industry Institute (Łukasiewicz-PIMOT), Jagiellonska 55 Street, 03-301 Warsaw, Poland

**Keywords:** vehicle passive safety, road traffic crash analysis, crashworthiness

## Abstract

The Rear Underrun Protective Device (RUPD) is a basic means to prevent a passenger car from running under the rear of a motor truck (also referred to as heavy goods vehicle or HGV) or a trailer in the case of a rear-end collision and thus to reduce deformations of the car’s passenger compartment (“survival space”). In many publications dealing with such devices, the increasing of RUPD stiffness by applying innovative design solutions or using high-strength materials has been considered; in some designs, additional RUPD components are introduced to absorb the impact energy. In this paper, a review of the RUPD designs is presented and some of them are analyzed, where their characteristics that are essential for the compliance with normative market requirements are indicated. Results of the authors’ research on the selection of an energy absorber incorporated in the rear impact guard bar of an HGV are presented as well.

## 1. Introduction

Over 1.3 million people are killed and another 20–50 million are seriously or severely injured every year in road traffic crashes all over the world, according to WHO [1]. The consequences of road traffic crashes constitute both a social and economic problem. In 2018, the unit cost per death and seriously or severely wounded in Poland amounted to PLN 2.4 and 3.3 million, respectively [2]. Therefore, the improvement in road traffic safety is an important issue for vehicle manufacturers, research, development, and scientific institutions, as well as governmental and non-governmental organizations worldwide.

Over the past 41 years, the number of passenger cars in Poland has increased over nine times. Over the past 11 years, growth has been 45% for passenger cars and almost 28% for trucks. This is an important factor in terms of road traffic intensity as well as the number of crashes [3,4].

According to the data published by the Polish Police Headquarters (Table 1) [3], the rear-end collisions, i.e., collisions where a vehicle crashes into the one in front of it, are Poland’s second most frequently occurring type of vehicle collision. The most dangerous collisions of this kind include those where a passenger car hits its front on the rear of a motor truck (also referred to as heavy goods vehicle or HGV). In such collisions, around 85% of passenger car go under the truck and the passenger compartment is damaged [5,6,7].

The available data on road traffic crashes in Poland do not provide unequivocal information about the number of collisions where a passenger car ran into the rear of an HGV. In the USA, however, 880 road traffic crashes, on average, defined as rear-end collisions with HGVs were annually recorded in 2015–2019, according to the FARS database [8,9]. In those collisions, more than 5000 people were killed. Moreover, it is known from the data of the Insurance Institute for Highway Safety (IIHS) in the USA that in 2018, 481 people were killed as a result of a passenger car’s impacts against the rear of a motor truck, which accounted for 23% of the victims of collisions between HGVs and passenger cars [10]. Rear-end collisions constitute 6% of lethal motor vehicle road traffic crashes in the USA; similar to Poland, they are one of the most frequent types of road collisions.

From the motor traffic safety point of view, two important goals should be considered here. The first one is to reduce the probability of a crash (active safety) and the other one is to minimize the car crash effects (passive safety). The level of passive safety of a vehicle chiefly depends on vehicle’s design features and on its additional equipment, e.g., safety belts or airbags. For every vehicle, it is important not only to ensure maximum safety for its occupants but also to guarantee adequate safety for other road users. Therefore, additional normative requirements are introduced in many countries, aimed at ensuring adequate safety for the unprotected road traffic users and, in the case of motor trucks, at the protection of passenger car occupants [11].

Over the years, passenger car manufacturers continuously modernized the vehicle design and introduced numerous engineering solutions to improve the safety of car occupants. Nevertheless, a collision of a car with a motor truck poses a high risk to the car users. Therefore, it is very desirable to reduce the hazards posed by HGVs to car occupants.

The Rear Underrun Protective Device (RUPD) is a basic means to prevent a passenger car from running under the rear of the bodywork of a motor truck, trailer, or semitrailer and thus to preclude or reduce deformations of the car’s passenger compartment (“survival space”). In this paper, a review and analysis of selected RUPD designs is presented. Special attention is paid to the fact that such devices, apart from their basic function, i.e., preventing a passenger car from running under the rear of the motor truck or a similar vehicle, should additionally absorb and dissipate a part of the impact energy in order to minimize the loads on the car occupants. The aim of this work is to answer the question of whether the appropriate RUPD design and the correct selection of the stiffness characteristics of its structural components (concerning legal and technical requirements) can help to minimize the dynamic loads on the occupants of the vehicle hitting the rear of the HGV. Moreover, it is also important to consider if the current regulations are not a barrier to development and implementation of safer RUPDs.

In Table 2, the methodology of the prepared review is presented. In step one, the review of the literature was completed. Next, the patent databases and the websites related to topic of the rear underride protection device were reviewed.

The review was limited to selected world markets due to the fact that the results of the analyses were used in the authors’ further research work related to the modified RUPD project. The new device will be primarily intended for the European market. Additionally, the RUPD constructions presented in the publication were carefully selected in order to present solutions showing various paths in the search for the most effective solution and the related limitations.

## 2. Normative Requirements

Vehicle testing may be performed in different ways, depending on the adopted criteria and purpose of the research. In terms of the test object, the following categories might be obtained:

– complete vehicle tests;
– component units tests;
– individual parts tests [12].

Although, in general terms, the HGVs do not differ from each other significantly in their design, they are subject to different normative requirements, depending on specific markets. Therefore, some differences occur between vehicles designed for similar or even identical applications; the differences may stem from, e.g., the product set-up or the engineering solutions used that have an impact on the passive safety, depending on the country and continent where the vehicles are operated.

**Figure 1:**
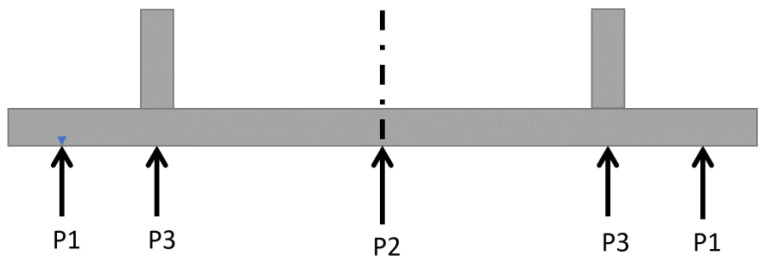
Schematic diagram of positioning the force application points during the RUPD strength tests.

Table 3 shows a summary of selected strength test requirements in force in various regions of the world, i.e., in Europe, Australia, USA, Canada, and China. When the RUPD test methods and requirements to be complied within selected world markets were compared with each other, an observation was made that there is similarity regarding the evaluation methods used. The basic RUPD test is the strength test with applying a load of up to 100 kN. Since 2017, the value of this maximum load has been raised to 180 kN in the European market and Australia, while in China, USA, and Canada, the RUPD is additionally required to have appropriate (but not identical) impact energy absorption properties. As an example: In the USA, pursuant to the governmental standard FMVSS [13,14], the RUPD should be subjected to a strength test, with the test force being applied at point P3 (Figure 1). When loaded with a test force of 100 kN and deformed by 125 mm, the RUPD should absorb an energy of 5650 J. In the Canadian market, the standard CMVSS 223 [15] shall apply, according to which the value of the energy absorbed by the RUPD is to be determined by applying a test force uniformly distributed along the whole bar of the test force application device. During the test, a force of 350 kN is applied and the minimum value of the energy absorbed should be 20,000 J. In China, in turn, not only a static strength test but also a crash test with an impact speed of 8.9 m/s (32 km/h) must be carried out, using a test carriage with a mass of 1100 kg representing a passenger car [16]. The test carriage deceleration measured during the collision shall not exceed 40 g and the carriage rebound velocity shall not exceed 2 m/s. This is to determine whether the test carriage impact energy can be effectively absorbed.

In the EU countries and Australia, the measurements of the energy absorbed by the RUPD are not required; instead, in consideration of the higher test load value prescribed (180 kN), structures of higher stiffness are designed so that the running of a passenger car under a motor truck is more effectively prevented. However, this may result in higher loads on car passengers during an impact against the rear of a motor truck [17,18,19].

Normative documents applicable, among others, to the USA, Canada, and China, indicate the max. test forces of 100 kN, which corresponds to the forces acting on the RUPD during the impact of a light passenger car on the rear of a truck, at an impact velocity of ca. 35 km/h. On the European market, the same requirements were in force until September 2017. New European standards require higher max. test forces (180 kN); thus, modern RUPD structures are intended to effectively protect against underrunning during the impact of a medium-class passenger car on the rear of a truck, for the impact velocity range of 40–45 km/h [20].

## 3. Analysis of the RUPD Designs and Test Results

**Figure 2:**
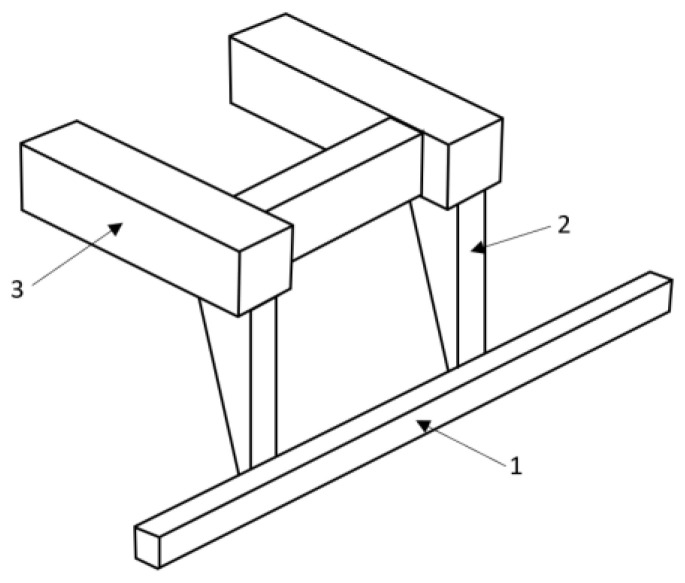
Standard rear underrun protective device. 1—RUPD guard bar; 2—Support member; 3—Vehicle frame.

A rear underrun protective device (RUPD) is fitted at the end of the truck body. It comprises a cross member beam and support members (usually two pieces). Most RUPDs are made of steel or aluminum and are fixed to a vehicle’s frame by screwing or welding. Based on the user’s requirement, the RUPD can either be a fixed or an adjustable device (e.g., foldable, slidable, and foldable–slidable). It depends on the specific purpose of the vehicle. The basic purpose of the rear underrun protective device is to minimize injuries to the occupants of the smaller vehicle (especially passenger car) in the case of a rear end collision with the truck or trailer. According to European law, all trucks should have a rear underrun protective device, suitable for a vehicle category, its weight, and load carrying abilities. A general layout drawing of the standard rear underrun protective device is shown below (Figure 2).

For the rear underrun protective device (RUPD) to function as required, it must have special constructional features. At the rear of a motor truck there is a big ground clearance under the truck’s load bed, which makes it possible for a passenger car to run into the space under the truck. Therefore, it is extremely important to place the RUPD in a correct position; the factors decisive for the RUPD performance are its ground clearance and distance from the truck bodywork. For the energy absorption capability of the car’s front structure to be utilized to the maximum extent and for the “wedge effect” (where the car front would slide under the RUPD and lift the truck’s bodywork) to be avoided, the ground clearance of the truck should never exceed 500 mm, preferably being close to 400 mm [21]. For the car’s penetration under the undercarriage of a truck or trailer to be minimized, the RUPD should be situated in the rearmost position, i.e., it should be roughly in the same plane with the rear end of the truck or trailer’s load bed. It has been stated in [22] that the RUPD should resist an impact of a medium-size passenger car (with a mass of 1200–1500 kg) moving with a speed of about 50 km/h and simultaneously withstand static loads of 100 kN when applied at P1 or P3 and of 150 kN when applied at P2.

In numerous publications dealing with the research on RUPDs, the raising of RUPD stiffness by applying innovative design solutions or using high-strength materials is considered. Moreover, the use of impact energy-absorbing components in some RUPD designs is additionally taken into account [22,23,24,25,26,27,28]. Selected RUPD designs and results from testing them are presented below.

**Figure 3:**
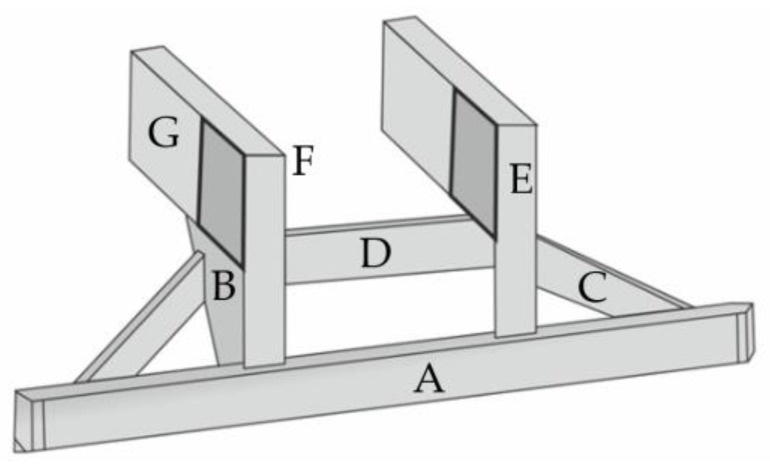
Hinged rear underrun protective device. A—cross-bar (guard bar), B—vertical supports (side surface), C—horizontal force supports, D—cross member, E—vertical force supports (end face), F—hinge, G—vehicle frame.

**Figure 4:**
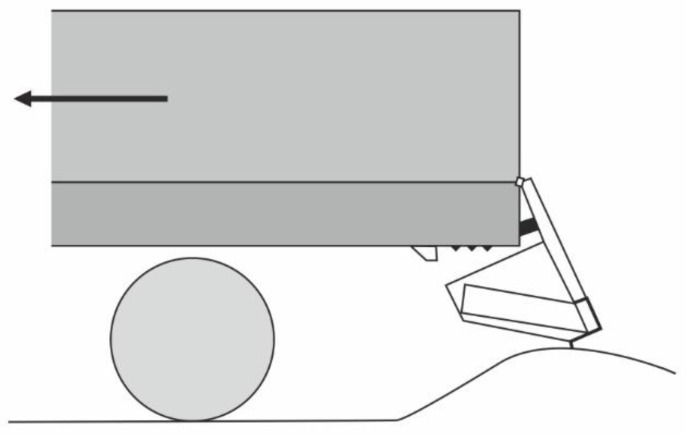
Principle of operation of the hinge used in the hinged RUPD.

A solution that makes it possible to ease the problem arising from a reduction in the departure angle in motor trucks is shown in Figure 3 and Figure 4 [22]. In a typical HGV design, insufficient RUPD ground clearance curbs the vehicle’s ability to move on roads with rapidly changing slopes. The solution proposed is a typical RUPD but fixed to the truck frame (G in Figure 3) by a hinge (point F in Figure 3). The hinge enables the RUPD to bend rearwards only; hence, when the truck is hit by a passenger car during a rear-end collision, the RUPD abuts against structural parts of the truck and cannot rotate around point F. On the other hand, when the RUPD bottom strikes against the ground, it bends rearwards without being damaged. Apart from that, support C connected with support B play the role of additional reinforcements of the end parts of the cross-bar (guard bar) (A in Figure 3). The RUPD design in question was based on the use of commercially available materials (steel profiles), simple installation on the vehicle, and minimization of device mass and manufacturing cost. The RUPD design basis was defined as follows: the device should resist an impact of a passenger car with a mass of 1200 kg moving with a speed of 50 km/h and simultaneously withstand static loads of 100 kN, when applied at P1 or P3, and of 150 kN, when applied at P2 (Figure 1). Alas, neither any test results nor strength computation results concerning the design presented were reported in the publication.

Based on an analysis of the information provided by the authors, it should be admitted that the hinge-type design is not in contravention of the regulations in force; hence, after the appropriate testing procedure is carried out and conformity with the applicable requirements is proved, the design might be approved for use in at least a few world markets. What remains to be clarified is the problem of durability of such a solution and possible damage to both the device and the road surface (scratching of the asphalt, paving blocks, etc.) when the device comes into contact with the ground.

**Figure 5:**
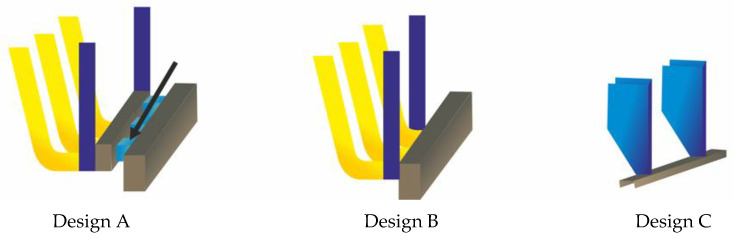
Schematic view of the RUPD versions used for tests.

Results of the tests carried out on another prototype rear underrun protective device (RUPD) have been published in [23]. The different versions of that device shown below (Figure 5) are provided with energy-absorbing components having the form of additional supports (Designs A and B). The version denoted as Design A has additional energy absorbers situated immediately behind the RUPD guard bar (and indicated by an arrow in the illustration). Design C is only a reference version used as a reference for comparative analyses of the test results obtained.

The computer simulations carried out by the authors using the LS-Dyna program represented the impact of a passenger car against the rear of a motor truck with speeds of 45, 54, and 63 km/h. The truck provided with the RUPD was fixed to the ground. The ground clearance of the RUPD was 450 mm. In each of the simulations presented, the RUPD was hit centrally.

The test results, i.e., results of measurements of the kinetic energy absorbed by the RUPD versions under consideration, are specified in Table 4. The results of measurements of the deceleration of the passenger car’s center of mass and of the maximum RUPD deformation are provided in Table 5.

An analysis of results of the simulations presented showed that the impact energy absorbed by the RUPD prototype built to Design A exceeded that absorbed by the reference version (Design C) by 68.8%. At the same time, the car deceleration dropped by 66% for Design A and by 41% for Design B, with the RUPD deformation being reduced accordingly. A comparison between the results of testing of Designs A and B reveals the influence of the additional energy absorbers. The energy absorbed by Design A exceeded that absorbed by Design B, with the deceleration value being simultaneously reduced by more than 30%.

Results of the successive development work and numerical computations carried out for the Design A presented above, where various cross-bar fillings were applied, have been reported in [24]. According to the tests, the lowest car deceleration value (12.8 g) has been recorded for the concept denoted by D (see Table 6). In consideration of the other parameters (energy absorption and deformation), the authors recognized the D concept as the optimum.

An analysis of results of numerical simulations showed that the system presented above effectively prevents the running of the passenger car under the rear of the motor truck. Furthermore, the values concerning the overloads that simultaneously occurred in the passenger car were reduced. However, given the lack of a validated numerical model, the system should be verified by carrying out a real crash test. The system presented above is not in contravention of the requirements of the normative documents described in the Section “Normative requirements”.

**Figure 6:**
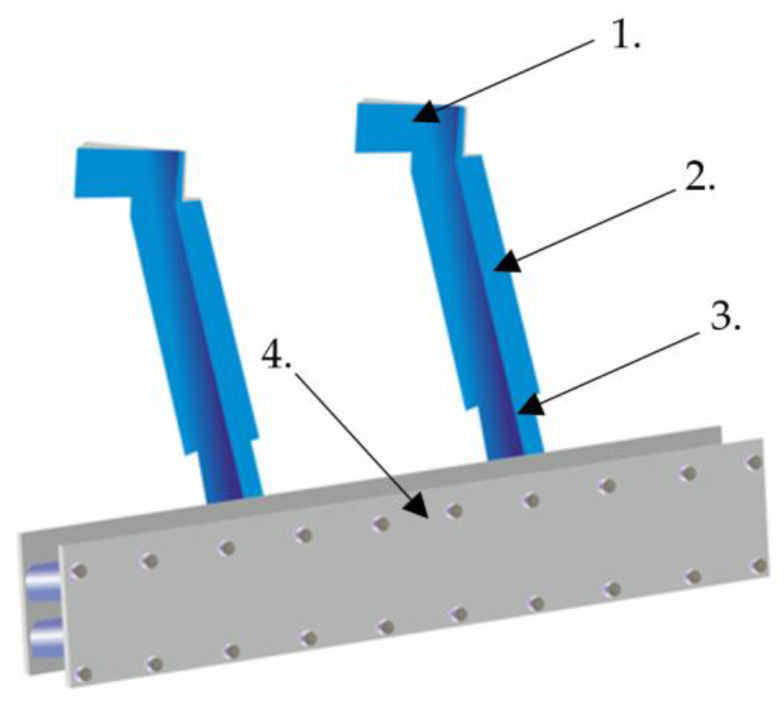
RUPD provided with impact energy-absorbing elements. 1—Support member fixed to the intermediate frame; 2—Support length adjustment mechanism; 3—Support member fixed to the RUPD guard bar; 4—RUPD guard bar with energy-absorbing elements.

A similar solution has been presented in [25]. Aluminum tube-shaped energy-absorbing structures are placed between two vertical walls of the RUPD guard bar (Figure 6). Moreover, the RUPD supports have adjustable lengths for the ground clearance of the guard bar to be changed as required.

**Figure 7:**
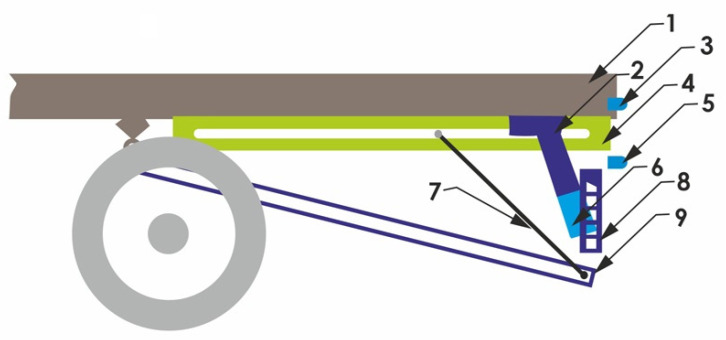
RUPD system for heavy goods vehicles. 1—Truck frame; 2—Support member fixed to the intermediate frame (4); 3—Speed sensor; 4—Intermediate frame of the system (support guide); 5—Distance sensor; 6—Support member fixed to the RUPD guard bar; 7—Lower platform holding-up ropes; 8—RUPD guard bar; 9—Lower platform.

Additionally, the RUPD cooperates with a supplementary safety system, which is shown in Figure 7. The principle of operation of the system as a whole is based on the cooperation of the passive part (RUPD proper, items 2, 6, and 8 in Figure 7) with the active part consisting of an additional lower platform (item 9 in Figure 7) and holding-up ropes (items 4 and 7 in Figure 7). When a rear collision hazard is detected, based on information received from sensors monitoring the speed and distance to the car following the motor truck (items 3 and 5 in Figure 7), the lower platform is lowered so that the car hits the RUPD guard bar and the platform holding-up ropes (item 7 in Figure 7). Simultaneously, at the instant of the impact, the upper rope anchorage points move towards the front of the truck and the lower platform is lifted to clamp the car’s front to prevent its further movement relative to the truck.

An analysis of results of numerical simulations showed that the system presented above effectively prevents the running of a passenger car under the rear of the motor truck. The simulation tests were carried out for two impact speed values, i.e., 13.9 and 22 m/s. The authors of [25] have stated that the stresses that occurred in the RUPD at the maximum deformation remained at a satisfactory level. Alas, no data on the overloads that simultaneously occurred in the passenger car during the computer simulations have been given. Moreover, in consideration of the system complexity and, in consequence, lack of a validated numerical model, the system should be verified by carrying out a real crash test. The system presented above is not in contravention of the requirements of the normative documents described in the Section “Normative requirements”.

**Figure 8:**
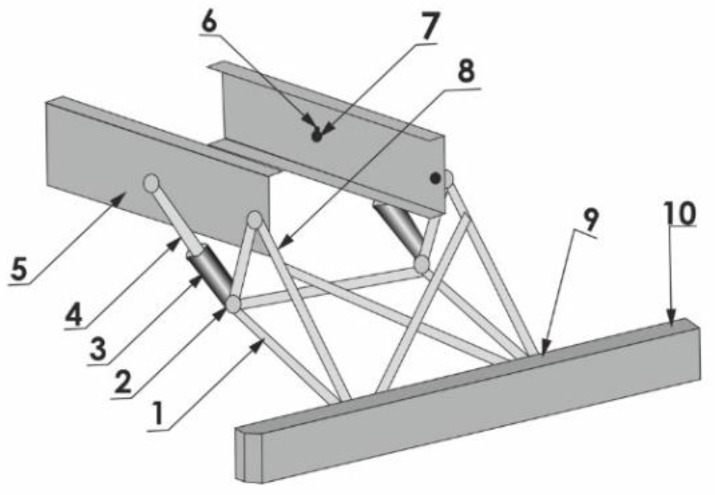
RUPD with energy absorbers in the guard bar and its supports. 1—Truss-form support system; 2—Pivotal joint between the energy absorber and the RUPD support; 3—Energy absorber; 4—Absorber arm; 5—Vehicle frame; 6—Cotter pin; 7—Pin; 8—Tapered stud bolt; 9—RUPD guard bar; 10—Energy-absorbing surface.

Another solution (Figure 8), presented in [26,27], was based on the use of supports made as a truss composed of tubular members (item 1 in Figure 8). To connect the supports with the truck frame (item 5 in Figure 8), an energy-absorbing member was additionally used (items 3 and 4 in Figure 8). As in the device shown in Figure 5, the RUPD guard bar (item 10 in Figure 8) is again an energy-consuming component. Thanks to such a design, the loads could be reduced by 40% in comparison with those developing in the case of the solution without any energy-consuming component.

When the possible applicability of this device to real HGVs is assessed, it should be stated that the engineering solution in question, if meeting the durability and dimensional requirements, might be approved for use in road vehicles, according to the regulations in force in all the markets previously described herein. In consideration of the current European durability requirements, however, it should be verified whether the solution in question can adequately resist test forces of up to 180 kN so that the maximum displacements are not bigger than the limits laid down in the UN ECE Regulation No 58.03 [18], i.e., 300 mm in the horizontal direction and 60 mm in the vertical direction, in particular because of the oblique position of the energy absorbers enabling both vertical and horizontal displacements of the RUPD guard bar (items 3 and 4 in Figure 8).

**Figure 9:**
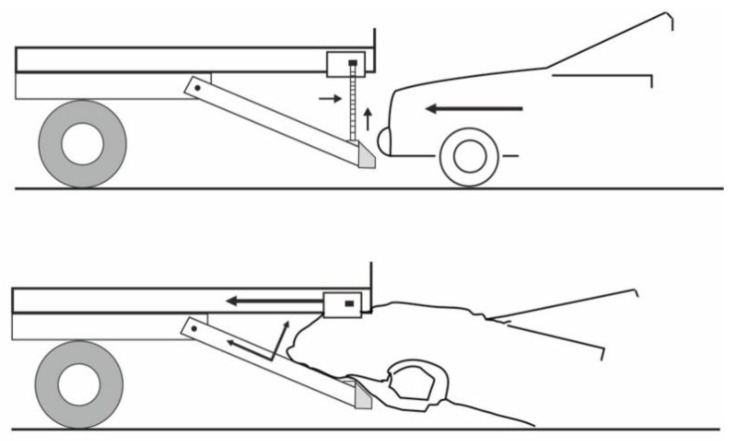
View of the RUPD named “Plier under-ride guard”.

An innovative RUPD design developed at the Biomechanics Engineering Laboratory, State University of Campinas (UNICAMP) in Brazil, has been presented in [26]. This unique design is known as “Plier under-ride guard” (Figure 9). The principle of operation of such a device may be described as follows. When a passenger car hits the device above the lower RUPD guard bar (situated below the lower edge of the car bumper), it gradually deforms a steel rope net situated between the lower and upper RUPD guard bars. The car front pressure onto the net causes the net ropes to pull the lower guard bar upwards and, thus, to restrain the car front. Results of a crash test, where a passenger car with a mass of 1490 kg hit the RUPD with a speed of about 64 km/h, have shown that the RUPD under test effectively prevented the car from running under the rear of the truck and, simultaneously, the loads on the dummy representing the car driver (Hybrid III) remained at an acceptable level. The maximum resultant acceleration of dummy’s head was 55.8 g and the HIC36 value was 381. However, the authors stated that, in spite of satisfactory results of crash tests, far-reaching modifications of the Plier under-ride guard are required from the commercial point of view, especially because of its mass and complicated construction, which affect its manufacturing cost. Due to its special principle of operation, such a system will not meet the current legal requirements. The basic problem arises from the hardly definable point of test force application during strength testing. According to the principle of system operation, the passenger car is to act on the steel net. Pursuant to the normative requirements, however, the RUPD deformation under load applied locally is to be evaluated during the tests. The requirements thus formulated are incompatible with the principle of operation of the Plier under-ride guard. Moreover, it is not unimportant, either, that the lower guard bar must move upwards during the impact, according to the principle of system operation; consequently, the ground clearance of the RUPD will increase with rising force acting on the system. Hence, such an engineering solution is in contravention of the requirements of European regulations.

**Figure 10:**
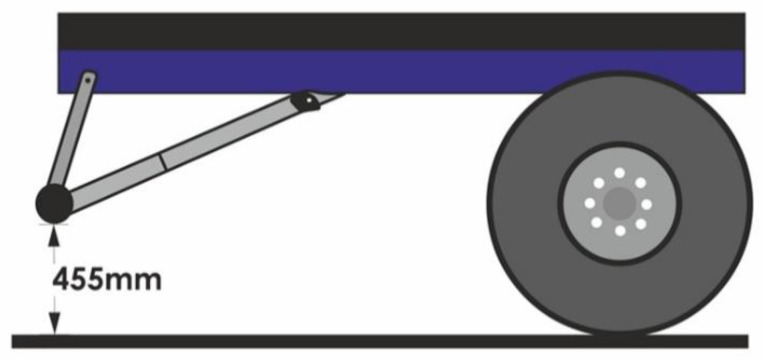
RUPD provided with a tubular energy absorber.

Figure 10 shows a solution where the RUPD guard bar is pin-connected to the vehicle frame. An additional energy-absorbing member is placed between the guard bar and the vehicle frame, obliquely to the frame. During an impact, the RUPD guard bar, guided by its swinging supports, moves towards the vehicle front, pushing against the energy absorbers.

The information provided in [26,28] is insufficient for the performance of this design to be more precisely evaluated, but the engineering solutions used here are not in contravention of the legal requirements applicable to RUPDs. However, the fact that the device in question must resist impact forces of up to 180 kN at relatively small vertical and horizontal displacements should also be taken into consideration. The absorber used, which supersedes the horizontal force support, would have to be characterized by a confined range of operation, lest its displacements should exceed the acceptable limits during tests. Moreover, the placing of the energy absorber pivot bearing far towards the vehicle front may constitute a difficulty for motor truck manufacturers because of the necessity of providing adequate space in the rear vehicle part.

**Figure 11:**
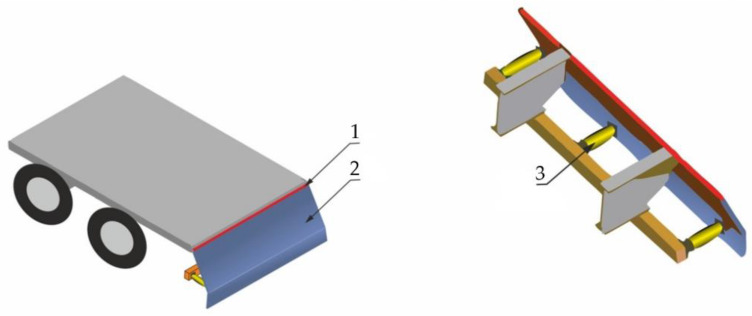
RUPD concept with a plate shielding the whole vehicle’s rear part under the cargo space. 1—Pivot; 2—Main Surface; 3—Energy Absorber (Crashbox).

A concept of an RUPD built in the form of a steel plate hinged on the rear part of a motor truck (Figure 11) has been presented in [29]. The plate is situated almost vertically, transversely in relation to the longitudinal vehicle axis. Its upper edge is fastened by pivotal joints to the vehicle frame and its lower part is supported by three impact energy absorbers (“crashboxes”). Each crashbox consists of an aluminum tube filled with metallic foam (Cymat A35620SC 030SS). The absorbers are attached to the plate (“main surface”) and the RUPD cross-bar by two ball joints; the cross-bar, playing the role of a support, is fixed to the vehicle frame. Simulation tests carried out have shown that such a solution makes it possible to reduce the impact loads on the passenger car at both the central and offset collision. The device effectively prevents the car from running under the rear of the truck, reduces the loads on car occupants, and limits the deformations of the car’s survival space. Actually, the new RUPD can absorb more energy than the standard one: by almost 90% in the case of a central collision and by about 20% in the case of an offset collision (where 40% of the width of the car’s front hits the RUPD).

The system presented above effectively prevents the running of the passenger car under the rear of the motor truck (for a small passenger car at impact velocity of 56 km/h). Furthermore, comparing with results of standard RUPD, the maximum peak of the measured decelerations has been reduced (averaged results). Nevertheless, it is worth noting that because of the greater stiffness of the side part of the proposed RUPD compared to the standard one, in the 40% overlap impact for some accelerometers, there is an increasing of the measured decelerations. As for above-mentioned constructions, the system should be verified by carrying out a real crash test, because of lack of a validated numerical model.

Although there are no unequivocal reasons, based on normative requirements, for the device described above not to be approved for use in vehicles, some aspects can seriously reduce its applicability. First, the mass of the RUPD in question exceeds that of most of the current solutions (by about 38%, according to estimates of its author) because of the necessity of using a plate of high stiffness. Moreover, a design similar to this precludes the installation of additional equipment, e.g., a truck tail lift or towing equipment.

**Figure 12:**
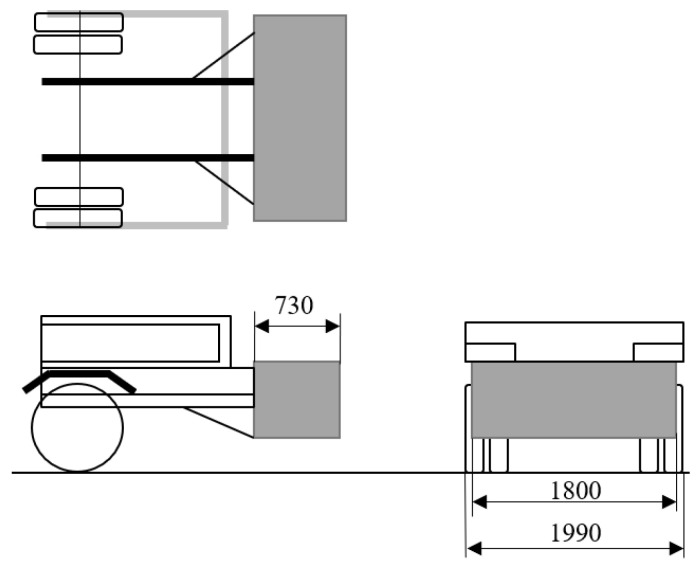
Impact energy absorbing device mounted behind the vehicle.

Another RUPD design similar to the one described above has been developed by the Truck and Bus Development of the Japanese Transport Department (Figure 12) [29]. This device consists of eight tubular energy absorbers placed between two plates at the rear of the truck. During a collision, the impact energy is absorbed by deformation of the tubes.

The entire device is installed behind the vehicle, which adversely affects the transport capability of the truck, including the cargo loading and unloading operations. Moreover, such an arrangement of the system precludes the installation of a tail lift or towing equipment and reduces the departure angle of the vehicle. Apart from that, the device of this type does not meet the normative requirement, according to which the RUPD should be situated as close as possible to the transverse vertical plane tangent to the rear extremity of the vehicle. Alas, no data on the overloads that simultaneously occurred in the passenger car during the computer simulations have been given. Thus, the evaluation of effectivity of that design cannot be given. Moreover, in consideration of the system complexity and, in consequence, lack of a validated numerical model, the system should be verified by carrying out a real crash test.

**Figure 13:**
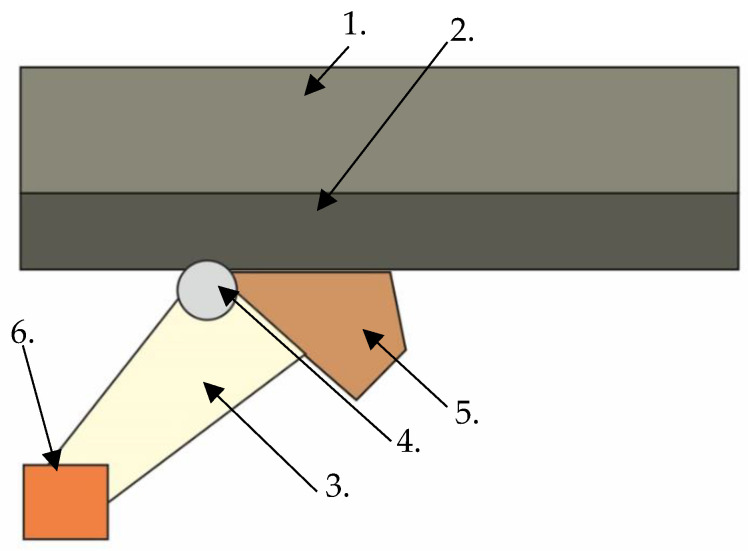
Rear underrun protective device RUPD-AB. 1—Part of the vehicle body; 2—Vehicle frame; 3—Support member; 4—Joint connecting support member with vehicle frame; 5—Energy-absorbing element; 6—RUPD guard bar.

In 2014, a RUPD concept provided with an additional energy absorber (named RUPD-AB) was presented by the company Kässbohrer [30]. It is based on the conventional RUPD design, where merely the supports were modernized by adding an energy absorber in the form of a wedge made of aluminum foam (Figure 13). Alas, there is no publication where any test results would be given or any information about the performance of the solution presented would be provided.

To express an opinion about the possible use of the device presented in a vehicle, it should be recognized that this engineering solution, if meeting the durability and dimensional requirements, might be approved for use in road vehicles, according to the European regulations. Alas, no data on the overloads that simultaneously occurred in the passenger car during the computer simulations have been given. As for the above-mentioned constructions, the system should be verified by carrying out a real crash test, because of lack of a validated numerical model.

**Figure 14:**
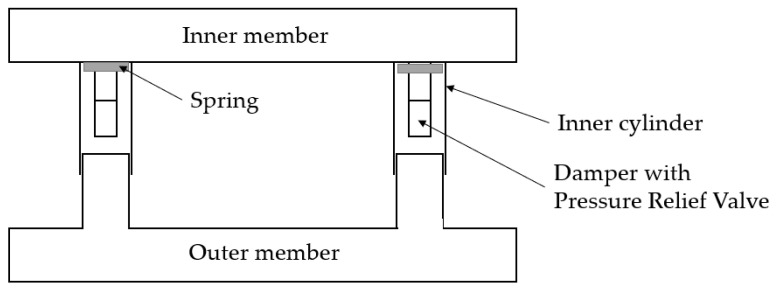
Block diagram of the rear underrun protection device with a pressure relief valve based damper.

Another solution of a rear underrun protection device has been presented in [31]. The concept based on the damper with a pressure relief valve is shown in Figure 14. The damper acts as a kinetic energy absorber and consists of an inner cylinder, a spring, and the pressure relief valve. The damper is located between the inner member (attached to the frame of the truck) and the outer member (RUPD beam and two brackets acting as pistons). The displacement of the pistons takes place when the vehicle is impacting the beam. Then, the impact force is transferred to the damper. As the inner member is attached to the chassis of the truck, the impact of the force on the HGV is negligible. Consequently, the inner member and HGV frame act as a rigid element. According to the authors, by implementing this RUPD it will increase the safety of passenger car passengers, but no analysis or test results have been presented that could prove the effectiveness of this device.

Based on an analysis of the information provided by the authors, it should be admitted that the rear underrun protection device with a pressure relief valve based damper is not in contravention of the regulations in force. After the appropriate testing procedure is carried out and conformity with the applicable requirements is proved, the design might be approved for use in at least a few world markets.

In article [32], the authors presented a similar device to that mentioned above, but instead of using a damper with a pressure relief valve, they fixed the crushing element. When impact force is applied on the outer member of the RUPD, the crushing element in the inner member is deformed. For reduction in impact force, the deformation of the crushing element plays an important role. In various scenarios (e.g., impact force varies), the number of crushing elements can vary. Static structural analysis was undertaken for this concept. The quantity of crushing elements to use was calculated and the maximum value of the stress evaluated. Unfortunately, the loads acting on passengers of a passenger car during the collision were not determined; therefore, the real effectiveness of the RUPD with a crushing element is undefined.

**Figure 15:**
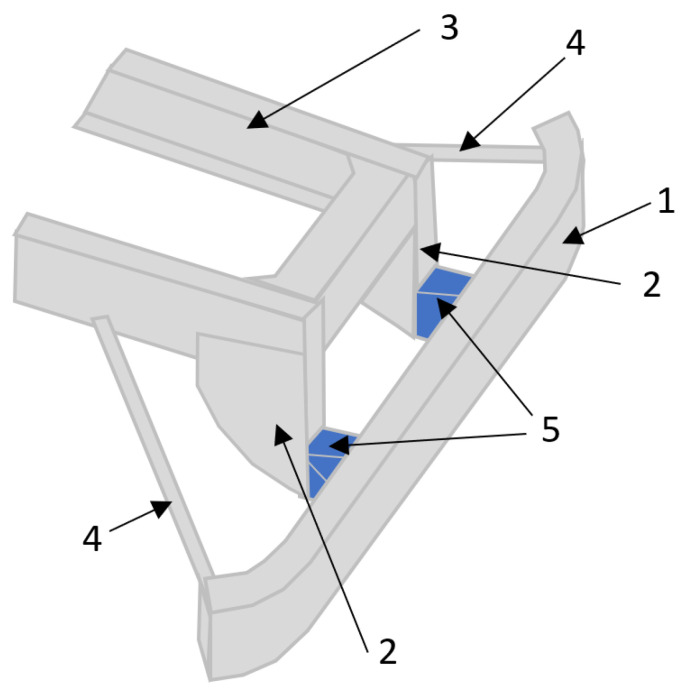
View of RUPD with enhanced energy-absorbing properties described in Patent Application No. DE102009036652A1. 1—RUPD guard bar; 2—Support members; 3—Vehicle frame; 4—Reinforcement bars; 5—Energy-absorbing elements.

In Patent Application No. DE102009036652A1 [33], an RUPD with enhanced energy-absorbing properties is presented. The invention was designed to be used in trucks. It has support members (item 2 in Figure 15) fixed to the vehicle’s frame (item 3) and reinforcement bars (item 4) supporting ends of the RUPD beam (Figure 15). The bars are designed as beams with a constant profile cross section over the length of the guard. Energy absorption elements (item 5) are placed between the barrier cross beam (item 1) and the support members (item 2). According to description in the application, the reinforcement bars might be made of extruded aluminum sheets or metal profiles in a rectangular or round tube shape. The inventors provided no details (e.g., shape, construction, materials) about energy-absorption elements; therefore, the effectiveness of the RUPD with enhanced energy-absorbing properties is undefined. The invention presented in Figure 15 is not in contravention of the regulations in force. After the certification process, according to appropriate requirements, is proved, the design might be approved for use in at least a few world markets.

**Figure 16:**
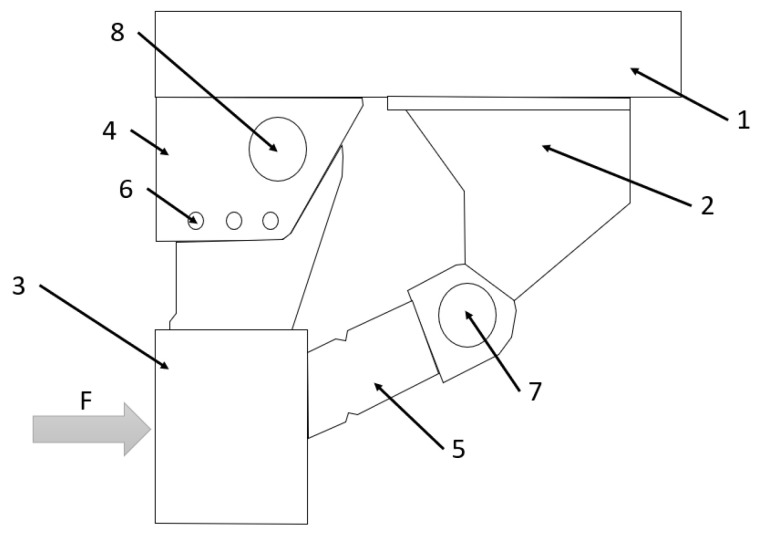
View of the RUPD with an energy-absorbing element described in Patent Application No. Patent EP1373025B1. 1—Vehicle frame; 2—Support frame; 3—Impact element with RUPD guard bar; 4—Support member for impact element; 5—Energy-absorbing element; 6—Locking element; 7, 8—Joints.

The next invention is described in Patent Application No. EP1373025B1 [34]. The invention should provide an improved underrun protection intended primarily for commercial vehicles (Figure 16). It comprises a frame (item 2), an RUPD guard bar ‘impact element’ (item 3) placed in a position that corresponds to an impact force direction F in the event of a possible collision with a vehicle, at least one support member-link element (4), which is firmly connected to the ‘impact element’ and it can pivot in relation to the vehicle’s frame (2), and an energy-absorbing element (item 5), which connects the impact element (3) to the frame (item 2) and is designed to be deformed due to a pivoting movement of the ‘impact element’ (3). The device comprises at least one locking element (item 6) to permit movement of an impact element only in the event of an impact with a force that exceeds a predetermined limit. There are no details about energy-absorption elements (e.g., shape, construction, materials) given in the above-mentioned application; therefore, the effectiveness of the described device is indeterminate. The same as the previous invention presented in Figure 15, the design is not in contravention of the regulations in force. Although this invention was designed to be mounted on the front of the truck, it might be approved for use as the RUPD, after the testing process according to appropriate requirements is proved.

**Figure 17:**
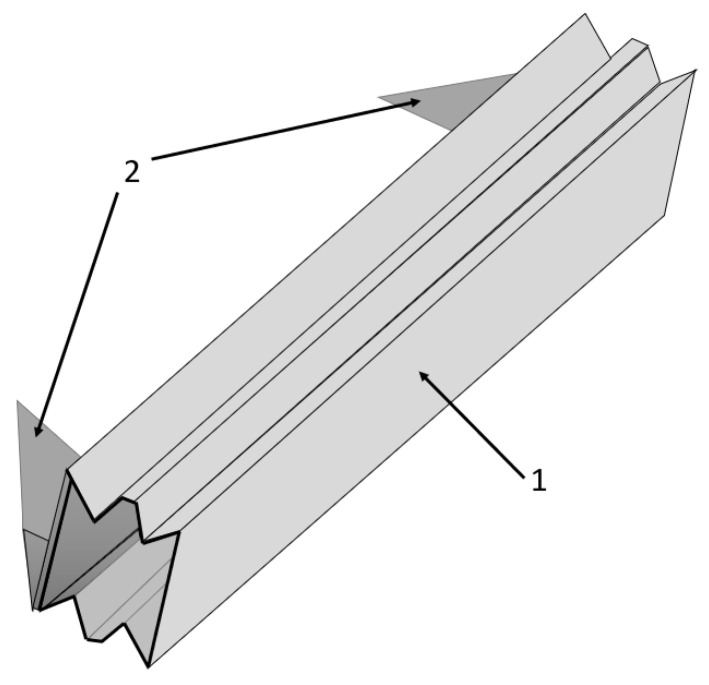
View of the RUPD structure based on a corrugated steel plate. 1—RUPD guard bar; 2—Support members.

Another solution of a rear underrun protection device is presented in [35] (Figure 17). The concept is based on a corrugated steel plate instead of the commonly used solid cross bar of RUPD in circular or rectangular cross sections. The RUPD with a cross bar made of corrugated steel plate is designed in a manner to absorb more impact energy and to offer more deformation. The same construction of RUPD was checked for different materials to esteem optimal performance (i.e., deformation) and energy-absorption capability. The authors also compared the results of FE static analysis with different models of a RUPD bar—the RUPD bar with a copper stiffener. As the authors stated, the RUPD with a corrugated structure has more deformation and energy-absorption capacity than the RUPD with copper stiffeners.

Based on an analysis of the information provided in [35], it should be admitted that the rear underrun protection device with a corrugated steel plate is not in contravention of the regulations in force. After the appropriate testing procedure is carried out and conformity with the applicable requirements is proved, the design might be approved for use in at least a few world markets. Unfortunately, FE analyses were undertaken according to IS 14812-2005 Standard; therefore, the value of the load was much lower than required by European regulation. In consideration of the current European durability requirements, it should be verified whether the solution in question can adequately resist test forces of up to 180 kN so that the maximum displacements are not bigger than the limits laid down in the UN ECE Regulation No 58.03 [18].

The analyses of the above-mentioned RUPD structures were carried out on the basis of the research results presented in the referred manuscripts. As the research and analyses of the described RUPDs were carried out by different research centers, the applied research methodologies differ from each other. Therefore, it is not possible to clearly indicate the most effective construction. Moreover, the publications do not present the results of experimental tests with real vehicles; therefore, the numerical models should be validated to make the results of the numerical analyses more reliable.

## 4. The Idea of an Energy-Absorbing RUPD

The design of a rear underrun protective device that would meet the applicable legal regulations, be inexpensive and easy to produce, and simultaneously be capable of absorbing the impact energy in a pre-planned and controlled way is a complex issue; on the other hand, it is also recommendable from the point of view of safety of passenger car users.

**Figure 18:**
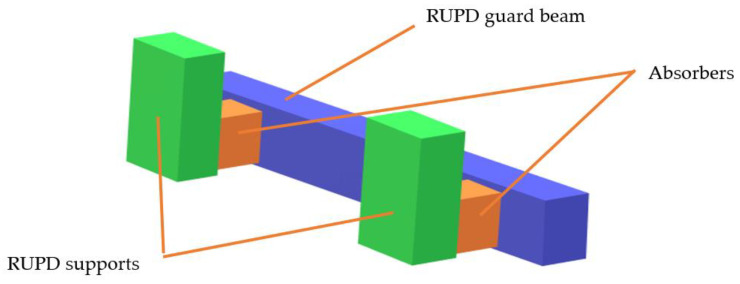
The idea of an impact energy absorbing RUPD.

The RUPD idea shown in Figure 18 includes elements of a conventional rear underrun protective device modified by the introduction of additional segments [36] accountable for the absorption of impact energy. The modification of this kind will ensure that the RUPD would meet the applicable legal requirements and only slightly increase the production costs (because of making use of the existing profiles and supports of the guard bar).

When the rear of a motor truck is hit by a passenger car with a mass of 1500 kg and a speed of 40 km/h (11.1 m/s), the desirable value of the energy absorbed is 93 kJ. The energy is mainly absorbed by deformation of the crumple zone of the car and deformation of the energy absorbers in the rear bumper of the truck. Hence, the more energy that is absorbed by the RUPD absorbers, the smaller the loads developed on the car.

**Figure 19:**
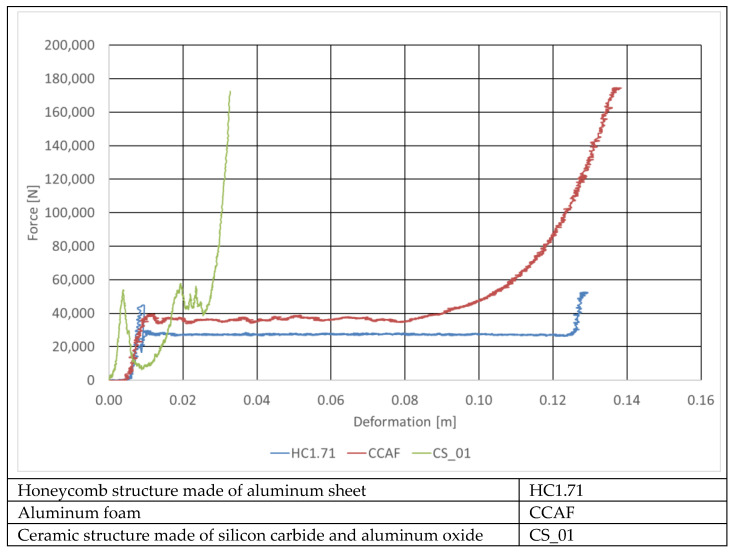
Stiffness characteristics of selected materials to be used as the absorber filling.

The absorber selection works were carried out by the authors of this paper on static and dynamic test stands at the Łukasiewicz Research Network—Automotive Industry Institute (Łukasiewicz-PIMOT). During the static tests, the stiffness characteristics of various absorber materials were determined (Figure 19). This made it possible to select the materials worth dynamic testing. As an example, the sample material whose force–deformation curve was plotted here in green was found unsuitable for being used as the absorber filling because it gets destroyed too rapidly, due to which its energy-absorbing properties are very much limited.

**Figure 20:**
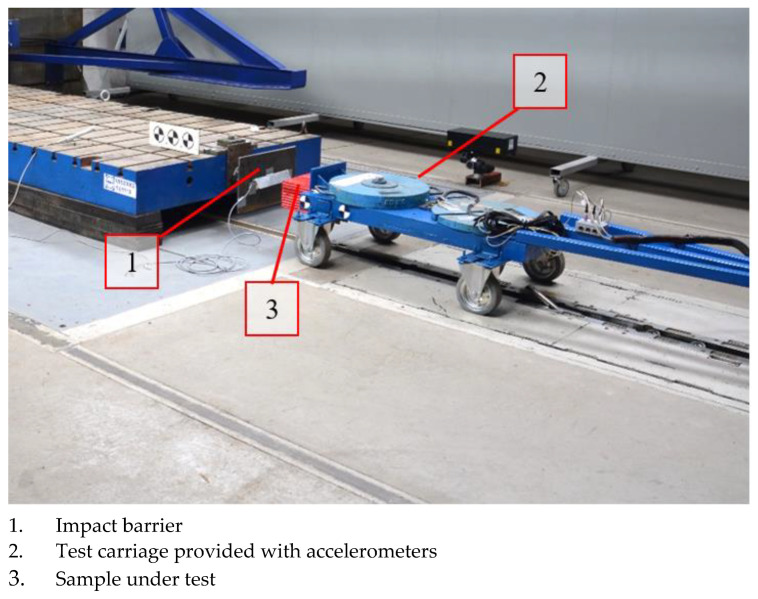
View of the dynamic test stand.

The energy-absorption characteristics of the samples selected were determined by carrying out impact tests using the test stand presented in Figure 20. For the tests, a carriage with a mass of 325 kg was prepared, to which the absorber under test was attached. The test carriage was accelerated to a speed of 5.44 m/s and hit a non-deformable barrier.

**Figure 21:**
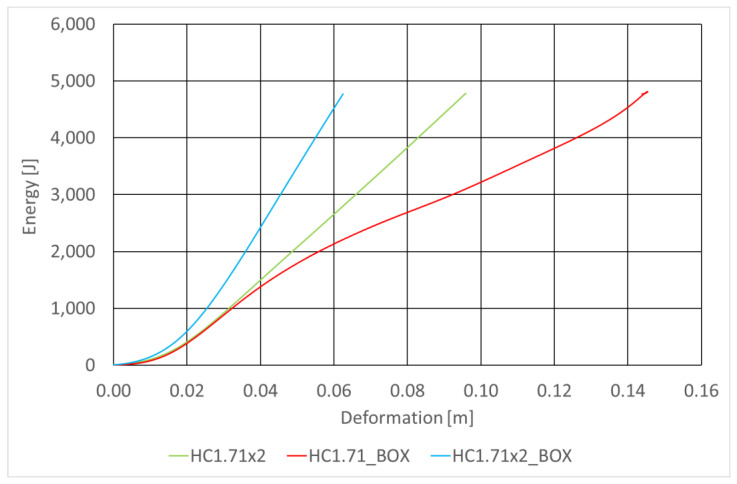
Comparison of the deformation values of the absorbers under test at the same impact energy.

**Figure 22:**
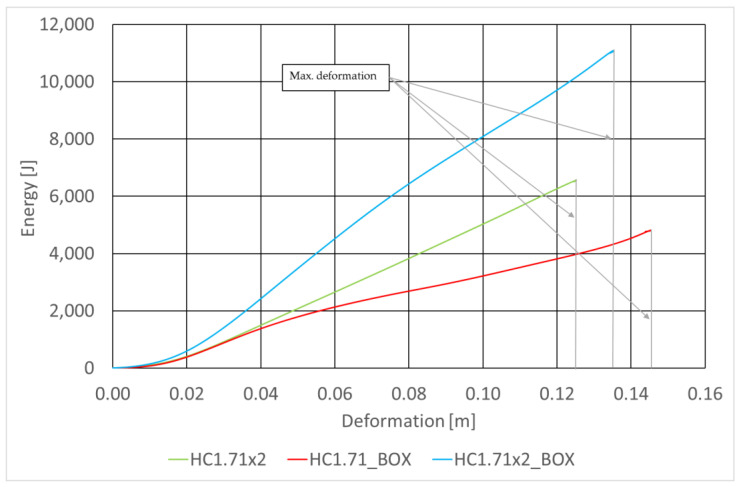
Energy absorbed at the maximum deformation of the energy-absorbing segments.

The basic absorber specifications are provided in Table 7 and Figure 21 shows curves representing the energy absorbed by the absorbers under test as functions of the deformation of the RUPD guard bar. The curves were plotted for the same impact speed of the test carriage, for absorbers mounted in box-type housings (BOX) with two values of width of the absorbing segment, to compare the absorbers with the one without a box-type housing. In order to estimate the space necessary to install the absorbers, their energy-absorbing capability was tested at the maximum (full) deformation of the absorbing segment. The test results obtained at the same initial thickness (12 cm) are summarized in Figure 22. According to expectations, the amount of the energy absorbed considerably depended on the absorber size (thickness). It was additionally noted that the mounting of the absorbing segment in a box-type housing significantly enhanced its absorbing characteristics. This may probably be an effect of increased friction between walls of the absorber and its housing; apart from that, this results from the stiffness of the housing itself.

The measurements showed that for the absorber material selected and the segment dimensions adopted, a growth in the energy absorption amount from 11% to 26% was achieved with the longitudinal deformation value remaining unchanged. Additionally, the post-impact carriage energy was determined during the tests; in each of them, this energy was practically imperceptible and did not exceed 1% of the pre-impact one.

**Figure 23:**
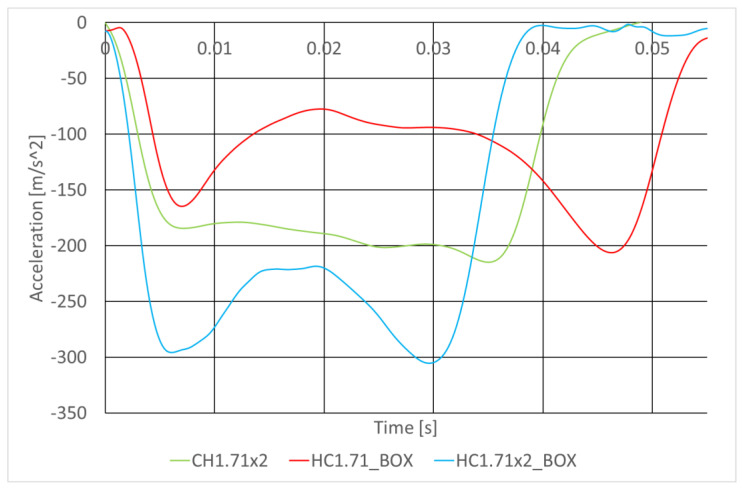
Acceleration vs. time curves.

Unfortunately, a growth in the absorber stiffness (due to a growth in the segment width and the use of a housing) resulted in a significant increase in the overloads on the impacting carriage (to 300 m/s^2^, i.e., by about 50%, see Figure 23).

**Figure 24:**
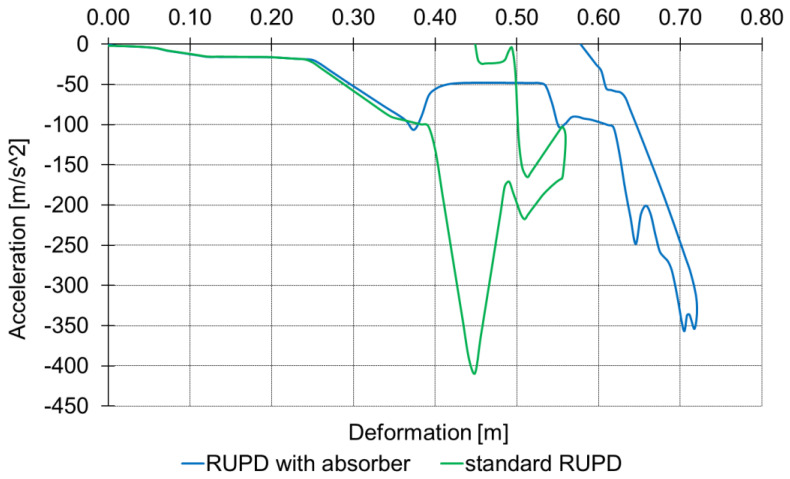
Results of computer simulation tests on the rear-end impact of a passenger car against a motor truck provided with a standard RUPD and an RUPD with an energy absorber.

In order to estimate the absorber’s performance in terms of the level of the overloads on a passenger car hitting the rear of the motor truck, computer simulation tests were carried out (in the Matlab-Simulink environment) [37,38,39]. Selected results of these tests are summarized in Figure 24.

The simulations revealed that the use of the HC1.71 × 2 absorber of 0.2 m length reduced the maximum deceleration of the passenger car by almost 13% and, in consequence, eased the overload on the car occupants.

## 5. Discussion

The review of requirements and engineering solutions as presented herein shows two basic types of designs of rear underrun protective devices (RUPDs): one in the form of a rigid structure capable of transmitting high loads and the other one being “flexible”, capable of absorbing the impact energy. In the European market, attention is chiefly focused on the solutions that can effectively prevent a passenger car from running under a motor truck or a similar vehicle. Therefore, the requirements applicable to RUPDs and being in force in the European Union and Australia were changed to increase the RUPD test load from 100 to 180 kN. This, however, involves additional stiffening of the RUPD structure and, in consequence, results in higher overloads on the car occupants during a rear-end collision with the truck.

In other world markets (China, USA, and others), the RUPDs are additionally required to absorb a definite part of the pre-impact energy of the motorcar. In this connection, new prototype designs are also being developed, which effectively lower the car occupant loads.

The vehicle manufacturers and research centers all over the world develop new RUPD designs. Some of the RUPDs presented herein are in contravention of the legal requirements currently in force; nevertheless, they make it possible to achieve a more effective protection of car occupants. Moreover, analyses of the described RUPDs were carried out by different research teams, so the applied research methodologies differ from each other. Therefore, it is not possible to clearly indicate the most effective construction.

The economic issue seems to be another barrier to the implementation of innovative solutions. For a newly implemented engineering solution to be not only in conformity with the applicable legal requirements but also competitive with other devices present in the market, it should be made of easily available and inexpensive materials. Until the use of energy-absorbing materials (in the European market) is forced by the normative requirements, the manufacturers will not be interested in introducing new solutions that are much more expensive.

The study showed that the appropriate RUPD design and the correct selection of the stiffness characteristics of structural components may be the solution to minimize the dynamic loads acting on the occupants of the vehicle impacting the rear of the HGV. Unfortunately, some of the current legal regulations might be a barrier to implementation of safer RUPDs. Hence, a way out may be the simple solution presented herein, which may be added to the existing rigid RUPD design with a low cost and labor input. It was shown in this paper that the introduction of a simple energy-absorbing segment makes it possible to reduce the maximum overload values by almost 15%. Due to current legal limitations, it is desirable to search for optimal (with limited effectiveness) solutions, but in the future it might be necessary to change legal requirements and ease implementation of more effective and innovative RUPD designs. Otherwise, the develop of safer RUPD might be difficult.

Moreover, to improve the safety of HGV, further work could include the proactive systems. To reduce the severity of the collision, those systems would react on the vehicle course and velocity. It might have a positive effect on the impact angle and velocity, in order to fully use of the RUPD features. Therefore, development of the active systems and real-time safety analysis might support the enhanced passive safety systems such as RUPD [40].

The authors are working on the selection of materials and construction of the absorbing segments to achieve even greater effectiveness of the absorption of impact energy. If the effects of the authors’ future work are satisfactory, the results might be implemented in real life by one of Polish trailer manufacturers.

## Figures and Tables

**Table 1:** Rear-end collisions in Poland.

Year	Road Traffic Crashes		Killed		Injured	
	Total	% *	Total	% *	Total	% *
2020	2676	11.4	179	7.2	3179	12.0
2019	3837	12.7	197	6.8	4751	13.4
2018	3998	12.6	207	7.2	5177	13.9
2017	4299	13.1	151	5.3	5676	14.4
2016	4267	12.7	165	5.5	5626	13.8
2015	3985	12.1	211	7.2	5108	12.8
2014	4218	12.1	206	6.4	5540	13.0

* Percentage of the total number of road traffic crashes, killed, and injured in the specific year.

**Table 2:** Methodology of the review (step-by-step).

Step 1—Review of the Literature	
Databases	Keywords
Scopus, Springer, Taylor and Francis, Web of science, ScienceDirect, JCR, IEEE Xplore, IOP Science, Elsevier, AIP, Emerald, BazTech	underride protection, underride guards, underride collision, all-round crumple zone, underride bumper, under run protection, rear underride guard, rear underride protective device, rear end collision, rear-end accident, RUPD
Step 2—Review of the Patents	
Databases	Keywords
Poland Patent Office (e- browser), Espacenet Patent search	underride protection, underride guards, underride collision, all-round crumple zone, underride bumper, under run protection, rear underride guard, rear underride protective device
Step 3—Review of the Websites	
Databases	Keywords
Google (accessed on 11 December 2021), www.nhtsa.gov (accessed on 11 December 2021), www.iihs.org (accessed on 11 December 2021), www.unece.org (accessed on 11 December 2021), www.policja.pl (accessed on 19 December 2021), www.wielton.com.pl (accessed on 19 December 2021), www.gniotpol-trailers.com (accessed on 19 December 2021), www.koegel.com (accessed on 25 December 2021), www.kaessbohrer.com (accessed on 25 December 2021), www.krone-trailer.com (accessed on 25 December 2021)	underride protection, underride guards, underride collision, all-round crumple zone, underride bumper, under run protection, rear underride guard, rear underride protective device, rear end collision, rear-end accident, RUPD

**Table 3:** Summary of the RUPD test conditions required in selected world regions.

Countries and Requirements	USA	Canada	Europe and Australia	China
Standard No.	FMVSS 223/224	CMVSS 223	UN ECE Reg. 58	GB 11567-2017
Height of the RUPD bar	min. 100 mm	min. 100 mm	min. 100/120 mm	min. 100/120 mm
Max. RUPD ground clearance	560 mm (before the test)	560 mm (before and after the test)	550 mm (before the test)	500/550/560 mm
Force applied at P1	50 kN	50 kN	100 kN	50 kN
Force applied at P2	50 kN	50 kN	100 kN	50 kN
Force applied at P3	100 kN	100 kN	180 kN	100 kN
Energy absorbed	5650 J (at P3), at 125 mm max. deformation	20,000 J (in the RUPD as a whole) at 350 kN	Not specified	Crash test with a test carriage Carriage deceleration shall not exceed 40 g

**Table 4:** Results of measurements of the impact energy absorbed, for 3 RUPD versions.

Test No.	RUPD Version	Impact Speed (km/h)	Energy Absorbed (kJ)
1	Design C	45	48
2	Design C	54	55
3	Design C	63	72
4	Design B	45	42
5	Design B	54	75
6	Design B	63	108
7	Design A	45	42
8	Design A	54	78
9	Design A	63	116

**Table 5:** Results of measurements of car deceleration and RUPD deformation, for 3 RUPD versions and various impact speeds.

Test	RUPD Version	Impact Speed (km/h)	Maximum Deceleration (g)	Maximum Deformation (mm)
1	Design C	45	34	927
2	Design C	54	41	1002
3	Design C	63	47	1091
4	Design B	45	20	327
5	Design B	54	21	509
6	Design B	63	24	678
7	Design A	45	14	204
8	Design A	54	15	409
9	Design A	63	16	638

**Table 6:** Concepts of the RUPD guard bar.

Guard Bar Type	Energy Absorbed (kJ)	Displacement (m)	Deceleration (g)
A—Steel profile without filling	87.2	0.077	38.4
B—Steel profile filled with aluminum foam	76.6	0.061	98.5
C—Steel profile with 14 rolled tubes	75.5	0.080	27.7
D—Steel profile with 14 rolled tubes filled with aluminum foam	74.8	0.080	12.8
E—Steel profile with 14 conical tubes	89.0	0.077	81.3

**Table 7:** Specifications of the energy-absorbing segments.

Designation	Description	Dimensions (m)
HC1.71 × 2	Honeycomb structure made of aluminum 3003 sheet, with a crushing strength of 1.71 MPa	0.24 × 0.12 × 0.20
HC1.71_BOX	Honeycomb structure made of aluminum 3003 sheet, with a crushing strength of 1.71 MPa, additionally reinforced with steel sheet walls	0.12 × 0.12 × 0.20
HC1.71 × 2_BOX	Honeycomb structure made of aluminum 3003 sheet, with a crushing strength of 1.71 MPa, additionally reinforced with steel sheet walls	0.24 × 0.12 × 0.20

## Data Availability

Not applicable.
